# Minimally Invasive Versus Open Total Hysterectomy: From Practice Variability to a Decision Algorithm

**DOI:** 10.3390/life16050749

**Published:** 2026-05-01

**Authors:** Nicoleta Alina Mareș, Alexandru Iordache, Niculae Iordache, Floris Cristian Stănculea, Ramon Vilallonga, Iuliana Ceaușu, Cristian Viorel Poalelungi

**Affiliations:** 1 General Medicine Faculty, Carol Davila University of Medicine and Pharmacy, 37 Dionisie Lupu Street, 020021 Bucharest, Romania; nicoleta-alina.mares@drd.umfcd.ro (N.A.M.); floris-cristian.stanculea@drd.umfcd.ro (F.C.S.); iuliana.ceausu@umfcd.ro (I.C.); cristian.poalelungi@umfcd.ro (C.V.P.); 2 Department of Obstetrics and Gynaecology, “Dr. I. Cantacuzino” Hospital, 030167 Bucharest, Romania; 3 “Professor Doctor Theodor Burghele” Clinical Hospital, 20 Panduri Road, 050659 Bucharest, Romania; 4 General Surgery Department, “Sfantul Ioan” Clinical Emergency Hospital, 13 Vitan-Bârzesti Road, 042122 Bucharest, Romania; 5 The Academy of Romanian Scientists, Ilfov 3 Street, 030167 Bucharest, Romania; 6 General Surgery Department, Universitat d’Andorra, Plaça de la Germandat, 7, AD600 Sant Julià de Lòria, Andorra

**Keywords:** laparoscopy, minimally invasive surgery, hysterectomy, benign gynecological pathology, clinical advancements

## Abstract

Background: Hysterectomy is one of the most common gynecological surgical procedures for benign conditions. In clinical practice, the choice of approach is often influenced by technology availability, leading to selection bias rather than to a decision based on standardized clinical criteria. The study aims to propose a standardized decision-making algorithm, based on objective clinical criteria, for selecting the surgical approach in total hysterectomy. Methods: This is a prospective, observational, analytical study conducted from November 2021 to June 2025, including a cohort of 332 patients who underwent total hysterectomy (LH, VH, AH) for benign gynecological pathology at two major centers in Bucharest. We proposed a decision algorithm based on the results obtained from predictive modelling and the quantified risk factors. Results: The choice of surgical approach is a critical factor in hematological stability, and the presence of comorbidities was associated with the type of approach (*p* < 0.001). Patients without comorbidities predominantly benefited from laparoscopy (48.85%), but as the anesthetic risk increased (ASA III and IV), the vaginal or abdominal routes were preferred. Conclusions: The convergence of these elements suggests that such a tool can standardize therapeutic decisions and improve the efficiency of interventions, offering a modern and predictable framework for gynecological surgery.

## 1. Introduction

Hysterectomy is one of the most common gynecological surgical procedures for benign conditions, with multiple approaches: vaginal, laparoscopic, abdominal, and robotic. In clinical practice, the choice of approach is often influenced by the surgeon’s skills, the availability of technology, or institutional preferences, which can lead to selection bias rather than to a decision based on standardised clinical criteria [1].

Currently, there is no standardised, internationally recognised score in gynaecology comparable to the nephrometric scores in urology, which have acronyms and are widely used to select the surgical approach in hysterectomy. However, various studies can serve as starting points for creating such a tool. As I summarised in a recent review, each technique presents distinct advantages and limitations regarding operative time, intraoperative blood loss, complication rates, recovery, or cost-effectiveness [2].

Some randomised studies from the United States have proposed protocols that consider the size of the uterus, its mobility, accessibility, and the presence of a pathology limited exclusively to the uterus (in the absence of adnexal conditions or suspicion of adhesions). Applying these criteria to select the most appropriate route for performing a hysterectomy has, in most cases, led to the use of minimally invasive surgery for benign conditions [3,4].

Additionally, ACOG and some Cochrane studies support the conclusion that, when feasible, vaginal hysterectomy yields better functional outcomes than abdominal hysterectomy, and laparoscopy offers significant advantages and is cost-effective compared with traditional surgery. These should be considered as the first-line approach in suitable cases [5,6,7,8].

In cases where a decision tree-based algorithm designated vaginal hysterectomy as the optimal approach, this option was associated with a shorter operative time, a lower incidence of postoperative infections, and minimal costs. Additionally, for more than 20 years, studies have shown that vaginal and laparoscopic approaches are associated with lower costs than abdominal hysterectomy, findings that may be useful for healthcare decision-makers [9,10]. Although laparoscopic procedures may be longer than VH, this technique is generally associated with a faster recovery, resulting in reduced analgesic use and a shorter average hospital stay. Additionally, in obese patients, the absence of parietal time during laparoscopy can reduce operative time compared with the open approach [11,12].

A thorough understanding of the benefits and limitations of each surgical approach is essential for making the most appropriate therapeutic decisions in patients undergoing hysterectomy for benign pathology.

Accurate case documentation and risk stratification are fundamental to ensuring quality standards and equity in the provision of gynaecological care services [13,14]. With this evidence, the present study aims to standardise clinical practice by minimising variability in the intraoperative selection of surgical approach and across centres. However, the decision regarding the surgical approach to hysterectomy must be made by the surgeon together with the patient, based on an assessment of the relative benefits and risks [7]. As analysed in a recent study, the choice of surgical approach is influenced by both the patient’s clinical status and the surgical team’s experience or individual preferences [15].

The aim of the study is to propose a standardized decision-making algorithm, based on objective clinical criteria, for selecting the surgical approach in total hysterectomy for benign gynecologic pathology and to evaluate its applicability.

## 2. Materials and Methods

### 2.1. Ethical Consideration

The analysis of the data in this study was conducted in accordance with the Declaration of Helsinki and approved by the Ethics Committee of “Dr. Ion Cantacuzino” Hospital (protocol code 13562/30 June 2025) and the Ethics Committee of “Sf Ioan” Clinical Emergency Hospital (protocol code R612/23 January 2026).

### 2.2. Study Design

This is a prospective observational analytical study that monitored, over time, variables that may influence the choice of hysterectomy approach, with the aim of establishing a standardised decision-making algorithm.

The study was conducted from November 2021 to June 2025 and includes a cohort of 332 patients who underwent total hysterectomy for benign gynecological pathology at two centres in Bucharest. The cases were selected from the Surgery Department of the “Sf Ioan” Emergency Clinical Hospital and the Gynaecology Department of the ‘Dr Ion Cantacuzino’ Clinical Hospital. Including cases from two different specialties is a strength of the study and provides an interdisciplinary perspective, ensuring a greater diversity of clinical situations and increasing the external relevance of the results.

### 2.3. Data Sources and Participants

The database included cases of hysterectomy performed via laparoscopic (LH), vaginal (VH), and abdominal (AH) approaches from the two centres that met the inclusion criteria and followed the proposed algorithm for the approach route. While various techniques stem from the basic methods, only those listed are currently practiced in these centers. In the future, the decision-making algorithm could also include vaginally assisted endoscopic surgery or robotic surgery.

The initial database, part of the author’s doctoral thesis, includes 961 hysterectomies performed for benign gynecological conditions. Data collection was prospective and approved by the Ethics Committee on 1 January 2021, with subsequent approval updates provided during the preparation of this article. Of these cases, 332 patients agreed to undergo surgery using the algorithm-suggested technique, which served as the study’s inclusion criterion. All patients signed informed consent. The remaining cases are subject to selection bias due to the surgeon’s preferences or hospital resources, which are outside the article’s focus.

Thus, a cohort of 332 patients was formed, comprising 135 laparoscopic hysterectomies, 102 classical surgeries, and 95 vaginal hysterectomies (Table 1). The hysterectomy procedures were performed with or without adnexectomy, unilaterally or bilaterally, depending on the indications, but this did not constitute a criterion for algorithm validation. Additionally, cases of prolapse were not mentioned to avoid influencing the trend toward selecting the vaginal approach.

The inclusion criteria were adult patients aged >18 years with a benign indication for total hysterectomy who provided consent to the algorithm-suggested technique. The exclusion criteria were malignancies, obstetric emergencies, or cases without consent.

The data were collected in a standardised manner to allow for objective processing. In addition to demographic data (age, BMI), patient characteristics at preoperative documentation were considered, such as uterine size, preoperative haemoglobin levels, comorbidities, and anaesthetic risk (the patient’s preoperative status was classified using the American Society of Anesthesiologists (ASA) Physical Status Classification), as well as clinical resources and the level of experience of the surgeons. Subsequently, a postoperative analysis was conducted, adjusting for postoperative haemoglobin level and average length of hospitalisation, to evaluate the approach’s effectiveness. All assessments were carried out by consensus to ensure consistency and minimise interpretive errors.

### 2.4. Working Protocol

The decision algorithm was developed using objective criteria informed by data from the literature. As working hypotheses, we started with the uterus size established in the guidelines as equivalent to 12 weeks of pregnancy, and then considered variables known to influence surgical technique, such as prior surgeries and comorbidities. The final stage was determined during the pre-anesthesia consultation, which assessed the patient’s risk and the optimal type of anesthesia.

The decision algorithm was created based on clinical criteria validated in the specialised literature for choosing the surgical approach in the case of hysterectomy. Each branch was established through consensus among gynaecological surgery experts, and the clinical thresholds were defined according to published guidelines and data, as described in the Discussion section. This algorithm is not a machine-learning model but a clinical tool.

The application procedure involved evaluating each case at admission, noting each component of the algorithm and the selected surgical approach. Subsequently, intraoperative and postoperative data were collected to assess the benefits of the selected intervention and the system’s applicability in clinical practice.

### 2.5. Statistical Analyses

Data processing and initial organization were carried out using Microsoft Excel and statistical analyses were performed using IBM SPSS version 23.0.

The distribution of data was evaluated using the Shapiro–Wilk normality test and the Kruskal–Wallis test. For paired variables that did not follow a normal distribution, the Wilcoxon signed-rank test was applied. Levene’s test was used to examine the equality of variances across different groups. To control for the influence of additional variables on the relationship between the main factor and the outcome, analysis of covariance (ANCOVA) was conducted. Categorical variables were analyzed using the χ^2^ (chi-squared) test, and statistical significance was defined as *p* < 0.05 for all tests.

## 3. Results

A decision algorithm is an organised set of steps and criteria for selecting the most appropriate option in a medical situation. In the case of a total hysterectomy, it helps the physician select the most appropriate surgical approach (abdominal, vaginal, or laparoscopic) based on the patient’s characteristics and the identified pathology.

The analysis of the study cohorts includes 332 hysterectomy procedures. The primary outcome was the selection of the surgical approach route. Overall, a preference for the laparoscopic approach is observed, used in 135 cases (40.66%), followed by the abdominal approach (102 cases—30.72%) and the vaginal approach (95 cases—28.61%). This distribution is highly statistically significant (likelihood ratio: 721.454; *p* < 0.001), reflecting a rigorous selection of cases based on the specific benefits of each method relative to the patient’s profile. In the case of hysterectomies accompanied by bilateral salpingo-oophorectomy (N = 234), the laparoscopic option remains dominant, followed by the abdominal approach (53.45% vs. 46.55%), due to the optimal visualisation of the lombo-ovarian ligaments, which are more difficult to access vaginally.

### 3.1. Analysis of Demographic Parameters

Patient age is the variable with the clearest statistical separation between the study groups. It was observed that patients subjected to the vaginal approach are significantly older, with a median of 68 years and a mean of 64.93 years (95% CI: 62.98–66.89). The negative skewness of age in the vaginal group (−0.738) confirms the clinical preference for this approach in the elderly population, likely related to the increased incidence of comorbidities and higher anaesthetic risks. In contrast, the abdominal and laparoscopic groups have much lower medians (51 and 49.5 years, respectively).

Regarding body mass index (BMI), the overall group has a mean of 26.78 kg/m^2^, which classifies them as overweight. It is worth noting that the laparoscopic approach was used across a wide range of BMI values, with an average of 34 kg/m^2^, contributing to both aesthetic outcomes and the reduction in potential complications related to difficult wound healing in obese patients. Demographic factors and population subgroups were thoroughly analyzed, but they were not relevant to improve the algorithm and are therefore not included in the paper.

### 3.2. Risk Profile: Comorbidities and ASA Score

The patients’ biological state exerted a critical influence on the therapeutic decision. Comorbidities and ASA score were statistically significantly associated with the type of approach (*p* < 0.001).

Patients without comorbidities (52.41% of the total) predominantly benefited from laparoscopy (48.85%), a trend also observed among patients with an ASA score of I, for whom laparoscopy was selected in 54.94% of cases. As the anaesthetic risk increases (ASA III and IV), the proportion of laparoscopic procedures decreases sharply, with vaginal or abdominal routes preferred. Clinical interpretation suggests a cautious approach: in patients with high biological risk, laparoscopy was avoided to limit cardiovascular and respiratory stress induced by pneumoperitoneum and prolonged Trendelenburg positioning.

### 3.3. Hematological Impact and Haemoglobin Dynamics

Dynamic, laparoscopic and vaginal approaches seem to offer an equally safe hematological profile, with the smallest average decrease in haemoglobin and the lowest postoperative standard deviation (1.32) (Table 2). In laparoscopy, this benefit is attributable to enhanced visualization, which enables direct and rapid haemostasis at the level of the vascular pedicles, thereby avoiding diffuse bleeding, compared with VH. Additionally, laparoscopy is the technique of choice for young patients, regardless of BMI or haemoglobin levels, offering a shorter average hospital stay, earlier recovery, and improved aesthetic outcomes.

To evaluate the impact of the surgical intervention on the hematological status across the entire group of patients (N = 332), a comparative analysis of preoperative and postoperative hemoglobin levels was conducted. The first step in the statistical validation process was to apply the Shapiro–Wilk. The result obtained (W = 0.941; *p* = 0.001) indicates that the distribution of hemoglobin differences does not follow a Gaussian (normal) curve. Clinically, this non-normality reflects heterogeneity in individual biological responses and variability in the treated pathologies.

Consequently, to compare the two time points, the Wilcoxon signed-rank test was used (W = 54,267.5; z = 15.378), indicating a highly statistically significant difference (*p* = 0.001) and confirming the hypothesis that hemoglobin levels decrease consistently following the surgical procedure. The descriptive data provide a clear picture of this transition, as shown in Table 3.

The multifactorial ANCOVA-type analysis used to determine postoperative hemoglobin levels is the most robust statistical model in the present study. Unlike previous models, Levene’s test for equality of variances yielded a *p*-value of 0.139, indicating homogeneity of variance and supporting the model’s high internal validity.

The surgical approach is statistically significant as an independent factor (*p* = 0.025), despite a relatively small effect size, indicating that, after accounting for the influence of age, preoperative BMI, and hemoglobin levels, surgical technique itself has a direct impact on the final hemoglobin level.

A particularly relevant aspect is the interaction between the surgical approach and the ASA score (*p* = 0.004; $\eta_{p}^{2}$ = 0.050). The choice of surgical approach (abdominal vs. laparoscopic vs. vaginal) becomes a critical factor for hematological stability, especially in patients with a high ASA score, where the capacity for compensation is limited. The model highlights complex interactions that extend beyond the simple addition of risk factors. Patients with comorbidities who develop postoperative anaemia experience much more severe drops in haemoglobin than each factor would suggest individually.

These data show that the postoperative hemoglobin level is not only a result of intraoperative bleeding but also the outcome of a complex interaction between the patient’s biological condition and the surgical procedure.

We conclude that preoperative optimisation of hemoglobin remains the safest strategy for preventing severe anaemia and that the prudent choice of surgical approach based on the patient’s profile is essential. However, a multivariate analysis would constitute a future study to clarify whether low hemoglobin levels (<10 g/dL) are a genuine determinant of the chosen approach.

**Figure 1:**
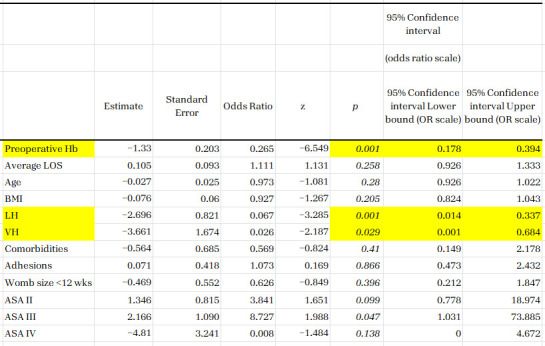
Logistic regression analysis, used to identify factors that significantly influence the likelihood of a medical outcome. The statistically significant predictors are highlighted in yellow, as described in the text: preoperative Hb, LH, and VH. The other variables did not reach statistical significance in this data set, but we kept them for data relevance.

The interpretation of variable effects in logistic regression models was performed using the odds ratio (OR), which indicates the extent to which each factor increases or decreases the likelihood of the outcome under analysis (Figure 1). The ORs reported for preoperative hemoglobin, the laparoscopic approach, and the vaginal approach reflect the association of these variables with the likelihood of developing anemia after the intervention, which is an important factor for selecting the approach route. The following results represent fundamental elements for the optimization of therapeutic protocols:

1. Preoperative Hemoglobin (OR = 0.265; *p* = 0.001): Each additional gram of hemoglobin at admission reduces the risk of postoperative anaemia by approximately 73.5% (1 − 0.265), which confirms that optimisation of the preoperative hematological status is the most effective prevention method.
2. Laparoscopic Approach (OR = 0.067; *p* = 0.001): Compared to traditional abdominal surgery, laparoscopy reduces the risk of anaemia by over 93%, demonstrating a clear superiority in preserving erythrocyte mass.
3. Vaginal Approach (OR = 0.026; *p* = 0.029): presents a high degree of protection, being an economical method in terms of blood loss.

### 3.4. Determinants of the Average Length of Hospital Stay

To identify the factors that independently influence the average length of hospital stay (LOS), an analysis of covariance (ANCOVA) was used. The model included LOS as the dependent variable and assessed the impact of surgical approach, postoperative anaemia, ASA score, prior surgeries, uterine size, and comorbidities, while controlling for continuous variables such as age, BMI, and hemoglobin levels.

**Figure 2:**
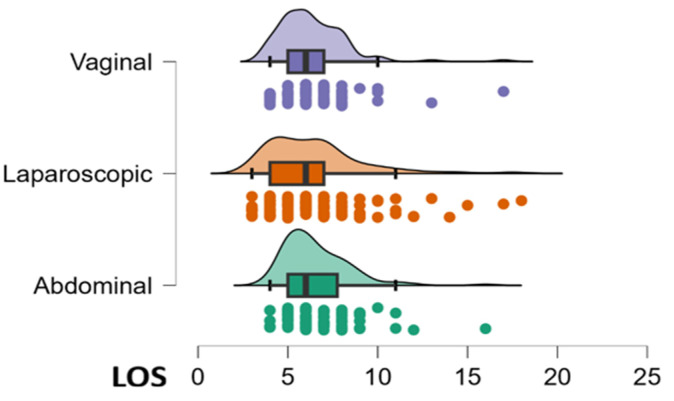
Distribution of LOS according to the surgical approach.

An innovative contribution to this model is the highlighting of statistical interactions, which show how certain factors influence each other, for example the association between the type of surgical approach and the size of the uterus (*p* = 0.001; $\eta_{p}^{2}$ = 0.048): a laparoscopic approach applied to a small-sized uterus results in a synergistic decrease in LOS, surpassing any other clinical combination (Figure 2). Additionally, a significant triple interaction was identified among postoperative anemia, uterine size, and patients’ comorbidities (*p* = 0.036), resulting in an exponential increase in hospitalization duration that exceeds the sum of the individual risks.

Modern management seems to neutralize the impact of age and BMI on LOS but remains dependent on the surgical approach and hematological stability. These two main factors appear to have a direct and quantifiable impact on the speed of recovery and discharge. Postoperative hemoglobin analysis (B = −0.378; *p* < 0.001) confirms that hematological stability is the primary condition for rapid discharge, and the vaginal approach (B = −1.036; *p* = 0.024) reduces hospitalization by more than 1 day (1.03 days), making it the optimal route for accelerated recovery.

It is remarkable to observe that the following factors did not significantly influence the duration of hospitalization, which suggests good standardization of surgical management:

• ASA score (*p* > 0.33): this indicates a high-performance perioperative care protocol that manages to compensate for pre-existing systemic risks.
• Intraoperative adhesions (*p* = 0.852): although they may prolong the actual operative time, adhesions do not affect the recovery duration, which emphasizes the optimal selection of the surgical approach route.

### 3.5. The Surgical Approach Path Selection Algorithm and the Predictability of Clinical Decision-Making

**Figure 3:**
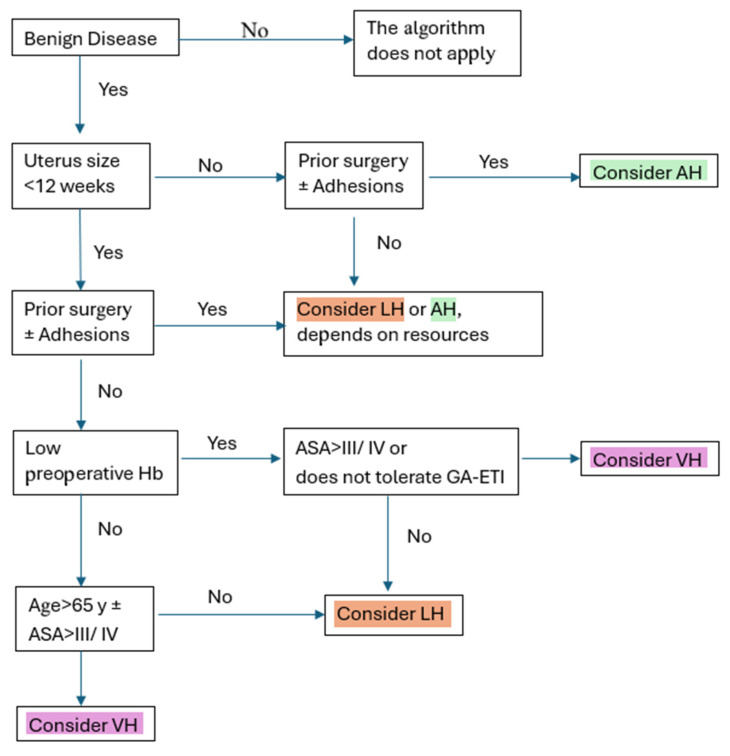
Documented surgical approach selection algorithm for total hysterectomy. The algorithm plays an advisory role and must be tailored to patients’ profiles and available resources to maximize benefits.

Based on the results of predictive modeling and the previously quantified risk factors, we propose implementing a documented approach path selection algorithm (Figure 3). The overall test accuracy of 64.6% indicates that the algorithm successfully captured meaningful and reproducible decision patterns; however, it does not fully capture the complexity of real clinical decision-making, such as a surgeon’s specific experience or current technical equipment.

The studied algorithm model indicates that the vaginal approach is predictable, while the laparoscopic technique directly competes with the abdominal approach for the same patient profile; the final choice is probably guided by medical nuances that are not statistically measurable.

The uterine size determines the access route; if it exceeds 12 weeks, laparoscopic or vaginal options are limited, favoring the abdominal approach for better exposure and safety. Hematological status influences technique choice; surgeons prefer ‘economic’ approaches like LH or VH for low hemoglobin to minimize blood loss. The ASA score and comorbidities guide decisions, especially since spinal anesthesia restricts access to vaginal methods. The surgeon’s experience and resources also matter.

To validate the algorithm’s accuracy, the classification error analysis (Confusion Matrix) provides valuable insights into areas with decision overlaps. A symmetric error rate of 0.12 between the abdominal and laparoscopic approaches indicates that some patients who could have benefited from laparoscopy still underwent open surgery. To measure the success of this algorithm, we propose monitoring the following key performance indicators (Table 4).

Implementing this algorithm not only enhances patients’ quality of life but also optimizes hospital resources by decreasing unnecessary hospitalization days and reducing the risk of hematological complications.

## 4. Discussion

The American College of Obstetricians and Gynecologists recommends minimally invasive surgery, and VH should be considered the first-line option whenever clinical and anatomical conditions allow, as the specialized literature consistently demonstrates better outcomes compared to other surgical methods. When the vaginal approach is not feasible or contraindicated, LH is advised, preferred over open abdominal hysterectomy due to its favorable recovery profile and lower morbidity [1,5].

Some algorithms proposed in the literature support decision-making based on clinical indicators, such as uterine size [12]. Several studies have examined factors influencing the choice of surgical approach in hysterectomy, but no standardized, easy-to-use scoring system for routine clinical practice has yet to be validated. We will now present the most relevant data from the literature that are consistent with our results.

The Indian triage system for vaginal hysterectomy, developed and described in a study published in the Journal of Obstetrics and Gynaecology of India, proposes a preoperative score to accurately estimate the feasibility of the vaginal approach in benign gynecological conditions. Its structure integrates essential clinical parameters, including uterine mobility and volume, with particular attention to cases exceeding the expected size for a 12-week pregnancy, the presence of adnexal formations, a diagnosis of endometriosis, and the identification of pelvic adhesions. Each element is scored, and the total score estimates the likelihood of conversion to an abdominal approach when vaginal hysterectomy cannot be performed optimally. By stratifying risk, guiding patient choice, and anticipating intraoperative challenges, this scoring system aids surgical decisions and improves outcome predictability [16].

A study published in Obstetrics & Gynecology evaluated the validity of guidelines for the prospective allocation of patients to vaginal, abdominal, or laparoscopic hysterectomy. The criteria used in this algorithm included uterine size, the presence of presumptive risk factors, and the degree of uterine or adnexal mobility and inaccessibility. The application of this system in clinical practice demonstrated its effectiveness in guiding patients towards the most appropriate surgical approach [11].

Another recent study from 2022 developed a scoring tool to evaluate laparoscopic hysterectomy complexity using standardized preoperative images, aiming to accurately predict the procedure’s technical difficulty. The final score, ranging from 1 to 4, indicates a progressive gradient of procedural complexity. Interobserver agreement was moderate, suggesting that the tool can provide useful preoperative guidance without replacing clinical judgment or the operator’s experience [17].

ISGE recommends vaginal hysterectomy as the preferred option for benign indications, if feasible, emphasizing the importance of the surgeon’s experience, vaginal accessibility, uterine size and mobility, as well as pathology limited to the uterus in the selection of patients for this method [18,19].

ACOG has retrospectively evaluated the usefulness of a decision algorithm based on vaginal accessibility and uterine size in guiding the surgical approach for hysterectomies performed for benign indications. The analysis highlighted that the systematic application of this algorithm optimized surgical route selection and resulted in significant institutional savings, thereby supporting its value in standardizing preoperative decision-making [11,20].

In 2020, at Mayo Clinic, a prospective algorithm was developed to optimize the selection of the surgical approach for hysterectomy based on three essential clinical factors: prior surgical procedures, uterine size, and vaginal accessibility. Using this algorithm has demonstrated the practicality of vaginal hysterectomy, showing a lower complication rate and favorable postoperative results.

Expanding its implementation could increase the proportion of total vaginal hysterectomies and generate significant savings for the healthcare system [21]. A crucial aspect remains the proper training of residents, for whom acquiring the specific skills and techniques of vaginal hysterectomy presents a challenge. Systematic integration of these skills into the training curriculum, alongside laparoscopic training, is essential to ensure the continuity and quality of minimally invasive surgical practice.

Promoting minimally invasive techniques requires a coherent strategy to compare the benefits and limitations of each approach, especially in situations where laparotomy remains the main approach for hysterectomy at many centers. In this context, a study proposed introducing a ‘technique index’, defined as the ratio of vaginal and laparoscopic hysterectomies to the total number of hysterectomies performed annually in a department. The authors also developed a scoring system to emphasize the advantages of minimally invasive methods and demonstrate the index’s usefulness in guiding clinical decisions and improving patient outcomes [22]. One study indicates that introducing vNOTES as an alternative to traditional surgery is practical and could improve peri- and postoperative outcomes [23].

Perceived technical difficulties, fear of complications, and the relatively low number of cases remain the main barriers to the widespread adoption of minimally invasive techniques in hysterectomy. For vaginal and laparoscopic approaches, limitations mainly stem from insufficient residency training, limited exposure to procedures, and the technical complexity specific to these methods, which often require longer surgery in the early stages of the learning curve.

Although most gynecologists claim they aim to reduce the number of abdominal hysterectomies in favor of minimally invasive procedures, clinical practice remains dominated by the abdominal approach, according to some studies from the United States [24,25]. This is likely due mainly to limited training and confidence among surgeons, rather than to the actual impossibility of minimally invasive methods. A clear generational gap is increasingly evident, with early-career doctors preferring laparoscopy, highlighting the need to improve training in minimally invasive surgery starting in residency.

Data from a German study indicate that adopting LH in routine practice is safe for patients and that increased surgical volume is associated with continuous improvement in technical performance. This progression is observed even among experienced surgeons who have performed more than 100 procedures, without any plateau in operative times, suggesting potential for sustained long-term refinement [26]. The surgeon’s experience in laparoscopic hysterectomy is essential for reducing the risk of complications, highlighting the importance of proper training for residents and gynecologists to perform this procedure with a high level of competence and safety, as all laparoscopic interventions require an optimal learning curve [27,28].

Minimally invasive approaches are safe and effective, associated with fewer complications, less blood loss, and shorter hospital stays compared to the abdominal approach [29]. Recent studies have confirmed that there are no significant long-term differences between vaginal and laparoscopic hysterectomy in quality of life and overall postoperative satisfaction, suggesting that both techniques can achieve similar functional recovery and patient well-being. However, from a socioeconomic standpoint, the laparoscopic approach seems to offer additional benefits, including shorter hospital stays and a quicker return to work, which can reduce indirect costs and have a positive overall impact on active life [30,31].

Over a five-year period, LH might be more cost-effective than VH, while offering similar benefits when technically feasible. The choice of surgical method should be based on a careful assessment of the specific risks and benefits for each patient, considering their clinical characteristics and the surgeon’s expertise [32].

More than the technique itself, optimizing perioperative management through measures such as intestinal preparation and integrated dietary control within the enhanced recovery concept facilitates rapid recovery after total laparoscopic hysterectomy, even in the absence of standardized protocols, at these two centers. This approach could reduce hospital stays, support incisional healing, and maintain an appropriate safety profile, according to a recent study analyzing the ERAS concept [33].

Although standardized algorithms can guide the choice of hysterectomy approach, careful patient counseling remains essential, as any surgical procedure involves unpredictable perioperative risks, regardless of the technique used. In this context, several recent studies have introduced multivariate logistic regression models to accurately predict the likelihood of major complications during laparoscopic or abdominal hysterectomies performed for benign conditions. These models include relevant clinical variables and generate personalized risk estimates tailored to each patient’s profile. This ability to individualize assessments provides valuable support for preoperative decision-making, promoting an informed and balanced discussion between the doctor and the patient [34,35,36].

The limitations of the study include the potential influence of each institution’s specific expertise and the variability of operator preferences across specialties, which may affect the distribution of approaches. One expected finding of the study is a variation in laparoscopy rates across centers, indicating either institutional preference or greater availability of expertise and equipment. The actual implementation of the recommended techniques can be influenced by individual choice, which may limit external generalizability.

This study does not meet the methodological criteria for algorithm validation; therefore, additional specific studies are needed.

## 5. Conclusions

This study addresses the need to standardize surgical approach selection for total hysterectomy by prospectively validating an algorithm to guide surgical decision-making.

The results demonstrate that the choice of approach routes is determined by both the patient’s characteristics (preoperative hematologic status, uterine size, previous surgical interventions, ASA score, comorbidities) and the institutional structure and the operator’s competence. Collectively, the results support the use of minimally invasive hysterectomy as a safe and effective approach for appropriately selected patients, including those with high-risk disease.

This algorithm is not yet a universally applicable clinical tool. Its widespread implementation requires additional multicenter validation across diverse populations and clinical contexts.

## Figures and Tables

**Table 1:** Proportion of hysterectomy cases according to the approach route.

Abdominal	Laparoscopic	Vaginal	Total
102	135	95	332
30.72%	40.66%	28.61%	100%

**Table 2:** Evaluation of the hematological reserves of the female patients included in the study and the average decrease in hemoglobin, depending on the surgical approach.

Surgical Approach	Preoperative Hb	Postoperative Hb	Average Decrease
Abdominal	12.35 g/dL	10.70 g/dL	1.65 g/dL
Laparoscopic	12.63 g/dL	11.02 g/dL	1.61 g/dL
Vaginal	12.56 g/dL	11.09 g/dL	1.47 g/dL

**Table 3:** Descriptive analysis of the entire group.

	N	Mean	SD	Coefficient of Variation
Preoperative Hb	332	12,528	1.66	0.133
Postoperative Hb	332	10,945	1.569	0.143

**Table 4:** Key Performance Indicators that measure the algorithm’s success.

Key Performance Indicators	Analysis-Based Target	Statistical Source
Postoperative Anemia Rate	<15%	Logistic regression showed that the minimally invasive approach could almost entirely prevent it.
LOS	<4 days	Linear regression showed that laparoscopic or vaginal approaches significantly reduce the current average of 6.4 days.

## Data Availability

The original contributions presented in the study are included in the article, further inquiries can be directed to the corresponding author.

## Abbreviations

The following abbreviations are used in this manuscript:

ACOG	American College of Obstetricians and Gynecologists
ASA	American Society of Anesthesiologists
LH	Laparoscopic Hysterectomy
AH	Abdominal Hysterectomy
VH	Vaginal Hysterectomy
BMI	Body Mass Index
Hb	Hemoglobin
LOS	Length of Hospital Stay
